# Heavy-slow resistance training in addition to an ultrasound-guided corticosteroid injection for individuals with plantar fasciopathy: a feasibility study

**DOI:** 10.1186/s40814-019-0489-3

**Published:** 2019-08-24

**Authors:** Henrik Riel, Jens Lykkegaard Olesen, Martin Bach Jensen, Bill Vicenzino, Michael Skovdal Rathleff

**Affiliations:** 1 Center for General Practice at Aalborg University, Fyrkildevej 7, 9220 Aalborg East, Denmark; 2 School of Health and Rehabilitation Sciences: Physiotherapy: Sports Injury Rehabilitation and Prevention for Health, The University of Queensland, St. Lucia, QLD 4072 Australia; 3 Center for Sensory-Motor Interaction (SMI), Department of Health Science and Technology, Faculty of Medicine, Aalborg University, Fredrik Bajers Vej 7D, 9220 Aalborg East, Denmark; 4 Department of Occupational Therapy and Physiotherapy, Aalborg University Hospital, Hobrovej 18-22, 9100 Aalborg, Denmark

**Keywords:** Plantar fasciopathy, Corticosteroid injection, Heavy-slow resistance training, Acceptability, Compliance

## Abstract

**Introduction:**

Plantar fasciopathy, characterised by plantar heel pain, affects one in ten in a lifetime. Heavy-slow resistance training (HSR) is an emerging treatment, but it often takes considerable time before the effect starts to manifest. Combining HSR with a corticosteroid injection (known for its short-term pain relief) could potentially improve outcomes in both short and long term. As this combination is yet to be investigated, we aimed to evaluate the feasibility of combining HSR with a corticosteroid injection for individuals with plantar fasciopathy before investigating the efficacy in a clinical trial.

**Materials and methods:**

We recruited 20 participants with plantar fasciopathy for this prospectively registered feasibility study (ClinicalTrials.gov: NCT03535896). Participants received an ultrasound-guided injection and performed heel raises on a step every second day for 8 weeks. To assess participant acceptability of the combined interventions and exercise compliance, we used a 7-point Likert scale dichotomised to “unacceptable” (categories 1–2) or “acceptable” (categories 3–7) and training diaries. Greater than or equal to 10/20 had to rate the combination “acceptable”, ≥ 15/20 had to perform ≥ 20 training sessions, and ≥ 15/20 had to start exercising ≤ 7 days after injection to confirm feasibility.

**Results:**

Eighteen out of 20 rated the combination acceptable. Five training diaries could not be retrieved. Ten out of 15 participants performed ≥ 20 training sessions, and 15/15 started exercising ≤ 7 days after injection.

**Conclusions:**

Based on participant acceptability and time to exercise start, combining HSR with corticosteroid injection is feasible and the efficacy should be investigated in a future trial. Due to loss of 5/20 training diaries, firm conclusions regarding exercise compliance could not be drawn.

**Trial registration:**

ClinicalTrials.gov, NCT03535896

**Electronic supplementary material:**

The online version of this article (10.1186/s40814-019-0489-3) contains supplementary material, which is available to authorized users.

## Background

Plantar fasciopathy is a common musculoskeletal condition and affects one in ten in a lifetime [1]. Pain is often exacerbated during the first steps in the morning and after prolonged periods of non-weight bearing [2]. Approximately half of patients referred to specialised clinics may still experience pain 10 years after treatment start [3]. Forty percent of patients still have symptoms after 2 years despite having performed plantar fascia-specific stretching and wearing insoles [4]. Patients with plantar fasciopathy have been found to show greater levels of depression, stress, anxiety, and kinesiophobia and have limitations in both mobility and health-related quality of life compared with sex- and age-matched healthy controls [5–7]. Plantar fasciopathy is also associated with several days of sick leave, and thus, plantar fasciopathy can have consequences for both patient and society [8, 9].

A recent systematic review and network meta-analysis compared the effect of several treatment options for plantar fasciopathy. It concluded that no single treatment was superior to others and that different treatments may take different time to work [10]. A corticosteroid injection has been found to be a safe option for plantar fasciopathy and has a good short-term effect compared with placebo, but there is no added benefit after 1 month [11–13]. One treatment option not included in the review was heavy-slow resistance training (HSR). HSR is a frequently used treatment option in the rehabilitation of both upper and lower limb tendinopathies and has also been found to be superior to stretching in plantar fasciopathy, but its effects usually take several weeks to manifest [14–17].

An injection and HSR could potentially supplement each other and provide the patient with both the immediate pain reduction associated with the injection and the long-term pain reduction from performing HSR. Repeated corticosteroid injections and a combination of stretching and strengthening exercises have been investigated before, but the combination of HSR and a single corticosteroid injection is yet to be investigated [18]. Due to the novelty of combining these two treatments, the feasibility should be investigated before investigating the treatment effect in a larger-scale trial.

The purpose of this study is to investigate the feasibility of combining HSR with an ultrasound-guided corticosteroid injection to reduce pain in individuals with plantar fasciopathy. Feasibility is evaluated using the acceptability of the combined treatments and exercise compliance.

## Methods

A cohort study design was implemented to follow patients with plantar fasciopathy over an 8-week period in order to determine feasibility of combining an ultrasound-guided corticosteroid injection with an HSR programme.

### Study design and setting

This study was designed as an interventional feasibility study. Reporting follows the items of the CONSORT 2010 statement: *extension to randomised pilot and feasibility trials* that are applicable to a non-randomised design [19]. Before the inclusion of the first participant, the study was registered on ClinicalTrials.gov (NCT03535896). All examinations were conducted at the Research Unit for General Practice in Aalborg, Denmark, by an experienced physiotherapist. Injections were performed at a private rheumatology clinic in Aalborg, Denmark, by a rheumatologist with more than 15 years of experience with ultrasound-guided injections. Data were collected using REDCap (Vanderbilt University, Nashville, TN, USA). Baseline and the 8-week follow-up were conducted at the study site whereas a link to the questionnaires used was sent by REDCap to participants’ e-mail address for the 4-week follow-up.

### Recruitment and eligibility criteria

Participants were recruited through social media (Facebook) or from a local general practice. The primary investigator performed telephone screenings of potentially eligible participants, and those who were not excluded based on this screening were invited to a clinical examination where final eligibility was determined. Inclusion criteria were as follows: (i) history of inferior heel pain for at least 3 months before enrolment, (ii) pain on palpation of the medial calcaneal tubercle or the proximal plantar fascia, (iii) thickness of the plantar fascia of 4.0 mm or greater measured by ultrasound [20], and (iv) mean heel pain ≥ 30 mm on a 100-mm VAS during the previous week. Exclusion criteria were as follows: (i) below 18 years of age, (ii) diabetes, (iii) history of inflammatory systemic diseases, (iv) prior heel surgery, (v) pregnancy or breastfeeding, (vi) corticosteroid injection for plantar fasciopathy within the previous 6 months, (vii) pain or stiffness in the first metatarsophalangeal joint to an extent where the exercises could not be performed, (viii) known hypersensitivity to corticosteroids or local anaesthetics, or (ix) skin or soft tissue infection near the injection site. These criteria were in line with those of similar studies in this patient population and had to be met by all participants [8, 11, 21].

### Intervention

#### Patient advice

After eligibility was confirmed, participants received information regarding what is known about the condition in terms of risk factors and aetiology, the pathology, and the rationale for why the combination of HSR and an ultrasound-guided corticosteroid injection could lead to recovery. They were advised to decrease activities which they felt caused symptom flare ups and slowly progress their activity level guided by symptoms. They were also informed about other types of evidence-based treatments; however, they were asked to refrain from seeking other treatments during the course of the study. Two weeks after inclusion, the primary investigator contacted participants to ask them if they had any questions regarding the condition or in relation to performing the exercise.

#### Heavy-slow resistance training and heel cup

Participants were instructed in performing a heel raise exercise standing with the forefoot on a step or a book as per Rathleff et al. [17]. The toes should be maximally extended by placing a rolled towel underneath them. Supporting themselves for balance by touching the hands on a wall or a rail was allowed. Participants were instructed to perform the exercise with a load as heavy as possible, but no heavier than they would be able to perform eight repetitions per set (i.e. eight repetition maximum (RM)) and for as many sets as possible. This self-dosed approach was found to be equal to the pre-determined programme used by Rathleff et al. [21]. Further information about the exercise is displayed in Table 1. If participants felt they were able to perform more repetitions than their load corresponded to (e.g. 10 repetitions when the load was supposed to be 8RM), an external load consisting of a backpack with books or water bottles to add weight was used. We told participants that pain during the exercise was expected and that there was no upper limit of pain they were allowed to experience as long as they felt it was tolerable. Participants were asked to start performing the exercise as soon as they felt ready but not before 24 h after the injection. During the first 2 weeks after the injection, they were asked not to progress the method used to achieve 8RM. If standing on both feet was sufficient to achieve 8RM at baseline, participants should not perform the exercise single-legged until the third week after the injection regardless of any pain reduction afforded by the injection. They were, however, still asked to perform as many sets as possible. Participants were told that complying with the exercise programme was very important and that exercise compliance was associated with their recovery. To support the exercise execution, participants received a training diary which included the exercise instruction and a link to a video in which the primary investigator showed the exercise instruction.

**Table 1:** Mechano-biological descriptors

1. Load magnitude	As heavy as possible, but no heavier than a weight that can be lifted at least 8 times (8RM)
2. Number of repetitions	≥ 8 depending on the load
3. Number of sets	As many as possible
4. Rest in between sets	2 min
5. Number of exercise interventions	Performed every other day
6. Duration of the experimental period	8 weeks
7. Fractional and temporal distribution of the contraction modes per repetition and duration (s) of one repetition	3 s concentric 2 s isometric 3 s eccentric
8. Rest in-between repetitions	No
9. Time under tension	8 s/repetition ≥ 64 s/set ≥ 64 s/training session
10. Volitional muscular failure	Yes
11. Range of motion	Full range of motion
12. Recovery time in-between exercise sessions	48 h
13. Anatomical definition of the exercise (exercise form)	The participant stands with the forefoot on a step. The toes are maximally dorsal flexed by placing a towel underneath them. The participant performs a heel raise to maximal plantar flexion in the ankle joint and afterwards lowers the heel to maximal dorsal flexion. Supporting oneself for balance by placing the hands on a wall or a rail is allowed.

A silicone heel cup was given to all participants, and they were advised to use the heel cup as much as possible. If participants already used an insole or any other type of foot orthosis, they were allowed to continue wearing this if they preferred it over the heel cup that we provided.

#### Ultrasound-guided corticosteroid injection

Participants received an ultrasound-guided corticosteroid injection between 5 and 8 days after baseline. A 21-gauge, 40-mm needle was connected to a 2.5-cm^3^ syringe filled with 1 ml triamcinolonhexacetonid (Lederspan, Meda A/S, Allerød, Denmark) + 1 ml lidocain 10 mg/ml (Xylocain, AstraZeneca A/S, Copenhagen, Denmark). The skin was cleansed with chlorhexidine alcohol 0.5% (Medic, Meda A/S, Allerød, Denmark). The needle was inserted with a medial approach under ultrasound guidance aligned to the long axis of the ultrasound transducer. The injection was placed anterior to the plantar fascia insertion on the calcaneal bone in the region of maximal fascia thickness.

### Outcomes

#### Feasibility outcomes

Before embarking upon a large randomised controlled trial investigating the treatment effects, it is recommended to investigate the feasibility including participant acceptability [22]. We chose three feasibility outcomes: (i) Acceptability of the combined treatments measured by a participant acceptability questionnaire that included a 7-point Likert scale ranging from “very unacceptable” to “very acceptable”. This was not a measure of whether participants’ symptoms had improved or not, but if the treatment matched their expectations to the content of the intervention and acceptability of performing exercises after receiving an injection. This was clearly stated in the questionnaire to emphasise that changes in symptoms were not to be considered. The combined treatments were categorised as “unacceptable” if they were rated as “very unacceptable” or “unacceptable” (category 1–2) and categorised as “acceptable” if they were rated from “slightly unacceptable” to “very acceptable” (category 3–7). We encouraged participants to elaborate their response in a free-text field. The questionnaire was filled out during the 8-week follow-up. (ii) Compliance to the exercises as measured by the mean number of training sessions performed throughout the intervention measured by a training diary that each participant is handed out at baseline. The participants were instructed in filling out the number of repetitions and sets performed and the day on which they performed the exercise. (iii) Mean days until participants started to perform the exercise measured from after the injection.

#### Explorative outcomes

In addition to the feasibility outcomes, we collected the following explorative outcomes to inform sample size estimations for a future trial: (i) change in the domains of the Danish version of the Foot Health Status Questionnaire (FHSQ) from baseline to the 4-week and 8-week follow-ups [23]. The FHSQ is a self-report questionnaire ranging from 0 (poor foot health) to 100 (optimum foot health) that assesses multiple dimensions of foot health and function and has a high reliability (ICC = 0.74–0.92) [24]. The minimal important differences of the domains are 14.1 points for pain, 7.4 points for function, and 9.2 points for general foot health [25]. (ii) Change in mean daily heel pain measured on an 11-point Numerical Rating Scale (NRS) (ranging from 0 which is no pain to 10 which is worst pain imaginable) from before the injection to 1 week after. This was chosen as an outcome to explore the short-term effects of the injection. The minimal important difference of an 11-point NRS is 2 [26, 27]. Participants received an SMS at the same timepoint every day in which they were asked to rate their mean heel pain during the past 24 h. The first SMS was sent the day after baseline, and SMSs were sent until 1 week after the injection. The SMS was sent using a smartphone (Huawei Y5, Huawei Technologies, Shenzhen, China) and the application Do It Later (Go Vap Dst, Ho Chi Minh City, Vietnam). (iii) Self-reported improvement measured on a 7-point Likert scale ranging from “much improved” to “much worse” (the Global Rating of Change (GROC)) at the 8-week follow-up. Participants were categorised as improved if they rated themselves as “much improved” or “improved” (category 6–7) and categorised as not improved if they rated themselves from “slightly improved” to “much worse” (category 1–5) [28]. (iv) Change in plantar fascia thickness from baseline to the 8-week follow-up measured in millimetres by ultrasonography. Measurements were performed using a longitudinal scan with participants lying prone with the toes placed maximally extended on the examination table. An average of three measurements was used (ICC = 0.67–0.77) [20]. (v) Change in self-efficacy as measured by the Pain Self-Efficacy Questionnaire (PSEQ). The PSEQ ranges from 0 (not at all confident) to 60 (completely confident) with lower scores indicating lower self-efficacy [29]. A validated Danish translation was used (ICC = 0.89) [30]. (vi) Change in physical activity level as measured by the International Physical Activity Questionnaire short version (IPAQ). The IPAQ estimates time spent performing vigorous and moderate activities, and time spent walking during the past week measured in MET-minutes [31, 32]. (vii) Recruitment rate defined as the mean number of participants recruited per week throughout the recruitment period.

The FHSQ, PSEQ, and IPAQ were filled out during baseline and at the 4-week and 8-week follow-ups whereas the GROC was filled out during the 8-week follow-up only. If participants were not categorised as improved based on the GROC, they were offered a second injection. If they accepted, they would receive the GROC again after an additional 8 weeks of performing exercises.

### Sample size

Due to the nature of a feasibility study, a formal sample size calculation was not performed [33, 34]. We aimed to include 20 participants as we considered this an adequate number to assess the feasibility of the combined treatments.

### Analyses

#### Feasibility

To conclude that the combined treatments were feasible, we a priori decided during a consensus meeting that the following three criteria would have to be met: (i) ≥ 10/20 rated the combined treatments as “acceptable”. If any participant dropped out after the injection, they would be dichotomised as “unacceptable”. (ii) Based on the self-reported training diaries, ≥ 15/20 participants would need to have performed ≥ 20/28 possible training sessions, and (iii) ≥ 15/20 participants would need to have started performing the exercise ≤ 7 days after the injection.

#### Explorative

We used histograms and Q-Q plots to assess data normality. Due to the nature of a pilot study, no hypothesis testing was performed and we report mean or median changes over time and 95% confidence intervals or frequency [19].

## Results

Recruitment was started on May 31, 2018. Between June 8 and August 10, 2018, we included 20 participants. The final day of data collection was October 11. Thirty-two potential participants were either referred from general practice or contacted the primary investigator directly. Of these, 24 were eligible for the clinical examination. Four were excluded; two individuals had a mean heel pain during the past week < 30/100 mm VAS, one individual was breastfeeding, and one individual had a plantar fascia thickness < 4 mm. After the final participant had been included, an additional 12 potential participants contacted the primary investigator to be included. One participant was lost to follow-up, and five training diaries could not be retrieved. None of these participants appeared dissimilar at baseline to those who handed in the training diary. Characteristics of the 20 included participants are shown in Table 2. One participant experienced an adverse event as she experienced pain in other areas of the foot than the heel during HSR. Participants' previous care-seeking behaviour is found in Additional file 1.

**Table 2:** Clinical and demographic baseline characteristics

Women (%)	16 (80)
Age (years)	51.7 (± 12.5)
Height (cm)	169.7 (± 8.9)
Mass (kg)	87.3 (± 16.3)
BMI (kg/m^2^)	30.3 (± 5.4)
Symptom duration (months)*	8 (6 to 11)
Pain during past week (/100 mm)	65.3 (± 13.3)
Bilateral pain (%)	6 (30)
Number of plantar fasciopathy episodes*	1 (1 to 3)

Data are presented as mean (SD) or count

*median (interquartile range)

### Feasibility results

The combined treatments were considered acceptable by 18/20 participants. According to the training diaries, 10/15 participants performed ≥ 20 training sessions (mean performed training sessions 20.8 (± 9.2)) and all started performing the exercise ≤ 7 days after injection (mean days 2.1 (± 1.1)).

Fifteen participants provided a reason for their response to the question of acceptability. The most common theme that emerged was reduced pain afforded by the injection when starting to perform the exercise (*n* = 3). Two participants thought that it was a good idea to combine several treatments to hopefully increase the odds of recovery. The one participant who evaluated the combined treatments as not acceptable reasoned this with increased pain in other parts of the foot than the heel when performing the exercise. All comments are found in Table 3.

**Table 3:** Participants’ reasons for their acceptability response. Translations were made as true to the original statement as possible

Original quote	English translation
Det har været super fint at være smertefri i startet, hvor jeg skulle påbegynde træning.	It has been super nice to be pain-free from the start when I had to start the training.
Grunden til jeg er meget enig er fordi, den første tid mærkede jeg ikke noget til smerterne pga. Injektionen hvilket gjorde det nemmere at gennemføre øvelserne og opgaverne i dagligdagen.	The reason why I very much agree is that from the beginning I did not experience pain because of the injection which made it easier to perform the exercises and everyday tasks.
Hvis det har en effekt og injektionen sker sjældent så finder jeg det acceptabelt og en god måde at komme videre på. Det er ikke just behageligt at få den, så vil selfølgelig helst undgå det. Men som sagt finder jeg det acceptabelt når man tænker på for og imod.	If it has an effect and the injection happens rarely then I find it acceptable and a good way of moving on. It is not necessarily comfortable to get it so I would, of course, rather avoid it. But, as I said, I find it acceptable when you consider the pros and cons.
Stadig smerter og kraftløshed	Still pain and debilitation
Virkningen af injektionen er udeblevet	The effect of the injection failed to happen
Ukompliceret og nem behandling.	Uncomplicated and easy treatment.
ikke mærket den store forskel, efter de 2 første uge	Not felt any big change after the first 2 weeks
Kombinationen gav mening. Der er enkelte gange gået mere end to dage mellem træningen.	The combination made sense. A few times it has been more than two days between the training.
Det værste var smerten i forbindelse med injektionen	The worst was the pain in connection with the injection
Meget fint med blot træning hver 2. dag, således ikke så tidskrævende.	Very nice with training just every two days so not that time consuming.
Øvelserne har givet voldsomme smerter andre steder i foden	The exercises have led to severe pains in other parts of the foot
Træningen blev langt nemmere og meget midre smertefyldt efter injektionen med binyrebarkhomon	The training became much easier and less painful after the injection with corticosteroid
Det kan siges acceptabel hvis der er nogen effekt af indsprøjtningen	It can be called acceptable if there is any effect of the injection
Om binyren har nogen effekt ved jeg ikke, med det at man HAR fået en sprøjte giver en vis “effekt” mentalt.	I do not know if the corticosteroid has any effect but the fact that you HAVE received an injection has somewhat of an “effect” mentally.
Godt med flere muligheder for behandling på en gang. Så større chance for at det virker.	Nice with more treatment options at once. So bigger chance for it to work.

### Explorative results

**Fig. 1:**
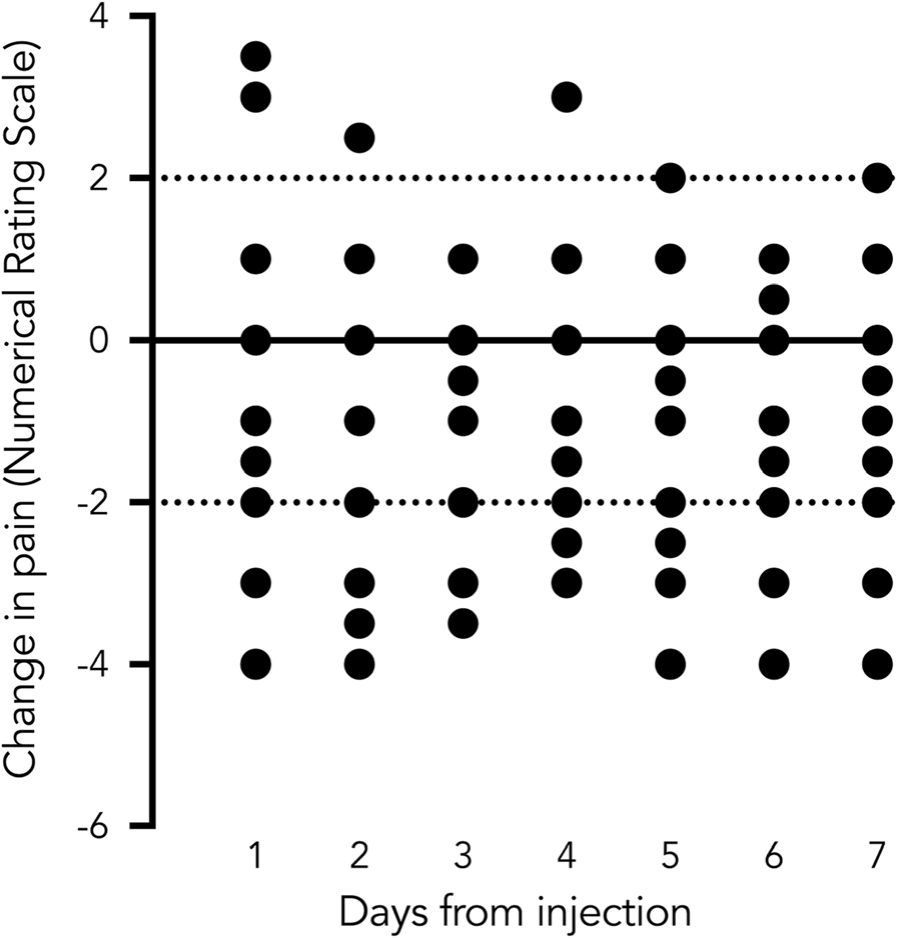
Individual changes in pain during the days after the injection relative to the median of pain each individual experienced during the days prior to the injection. Dashed lines show the threshold for a minimally important change on a 0 to 10 Numerical Rating Scale. Positive values are an increase in pain, and negative values are a decrease in pain

Participants improved from baseline to 4 weeks in FHSQ pain (mean change 15.8, 95% CI 3.0 to 28.6) and to the 8-week follow-up (mean change 13.5, 95% CI − 0.3 to 27.2). In the function domain of the FHSQ, participants improved the scores more than the minimally important difference of 7.4 points from both baseline to the 4-week follow-up and to the 8-week follow-up (Table 4). Mean daily heel pain decreased 1.2 NRS (95% CI 0.7 to 1.7) from the days before (mean pain 5.5 (± 1.8) NRS) to 1 week after the injection (mean pain 4.3 (± 2.1) NRS) (Fig. 1). According to GROC, 6/19 participants were improved after the intervention. Four participants of those who were not improved according to GROC agreed to receive a second injection. One was dichotomised as improved after the additional 8-week follow-up, and one still had not improved. The remaining two were lost to follow-up. We were actively recruiting participants for a total of 6 weeks which led to a weekly recruitment rate of 3.3 participants per week. The mean number of sets performed per training session was 4.2 (± 2.4).

**Table 4:** Results of explorative outcomes

	Mean (SD)	Mean change (95% CI)		
		Baseline vs 4 weeks	Baseline vs 8 weeks	4 weeks vs 8 weeks
FHSQ pain (0–100)				
Baseline	41.1 (12.7)	15.8 (3.0 to 28.6)	13.5 (− 0.3 to 27.2)	− 2.3 (− 12.2 to 7.6)
4 weeks	56.5 (26.6)			
8 weeks	54.8 (28.2)			
FHSQ function (0–100)				
Baseline	61.9 (19.3)	11.8 (− 0.1 to 23.7)	12.9 (− 1.4 to 27.1)	1.0 (− 9.8 to 11.9)
4 weeks	71.9 (24.8)			
8 weeks	74.3 (26.0)			
FHSQ footwear (0–100)				
Baseline	35.8 (21.8)	8.8 (− 5.0 to 22.6)	12.0 (− 0.4 to 24.5)	3.2 (− 2.6 to 9.1)
4 weeks	45.8 (29.0)			
8 weeks	48.3 (27.6)			
FHSQ general foot health (0–100)				
Baseline	44.5 (21.0)	− 6.3 (− 21.3 to 8.8)	9.0 (− 0.2 to 18.3)	15.3 (2.4 to 28.2)
4 weeks	35.1 (27.5)			
8 weeks	50.9 (26.6)			
PSEQ (0–60)				
Baseline	42.1 (8.9)	5.2 (0.5 to 10.0)	5.8 (0.2 to 11.3)	0.6 (− 4.4 to 5.5)
4 weeks	47.0 (12.2)			
8 weeks	48.2 (10.6)			
Plantar fascia thickness (mm)				
Baseline	5.6 (0.9)		0.3 (− 0.1 to 0.7)	
8 weeks	5.3 (1.2)			
	Median (IQR)	Median change (95% CI)		
		Baseline vs 4 weeks	Baseline vs 8 weeks	4 weeks vs 8 weeks
IPAQ walk (MET)				
Baseline	1155 (330–1732.5)	− 132 (− 251 to 231)	− 99 (− 921 to 317)	− 1155 (− 1598 to − 330)
4 weeks	1386 (198–2079)			
8 weeks	495 (297–1386)			
IPAQ moderate (MET)				
Baseline	540 (300–2220)	0 (− 1254 to 600)	0 (− 480 to 480)	600 (− 2104 to − 360)
4 weeks	720 (40–2880)			
8 weeks	480 (240–960)			
IPAQ vigorous (MET)				
Baseline	440 (0–1520)	0 (− 480 to 480)	0 (− 73 to 313)	− 400 (− 1107 to 0)
4 weeks	240 (0–1440)			
8 weeks	240 (0–960)			
IPAQ total (MET)				
Baseline	2475.5 (1391–4614)	242 (− 922 to 2681)	− 171 (− 1592 to 864)	423 (− 712 to 2084)
4 weeks	1935 (1200–6906)			
8 weeks	2217 (1059–2772)			

*FHSQ* Foot Health Status Questionnaire, *PSEQ* Pain Self-Efficacy Questionnaire, *IPAQ* International Physical Activity Questionnaire, *MET* metabolic equivalents

## Discussion

### Key results

This was the first study that included both HSR and an ultrasound-guided corticosteroid injection in the treatment of plantar fasciopathy. We found that 18/20 participants rated the combined treatments acceptable, and according to the 15 training diaries retrieved, they were adequately complying with the exercise programme.

### Interpretation of feasibility

Despite the efforts made to ensure that participants disregarded any changes of their symptoms in the evaluation of acceptability, several of the comments (6/15) concerned the treatment effect and differentiating between treatment effect and acceptability of the content of treatments appeared to be difficult. Even if only 6/19 participants improved according to GROC, nearly all participants evaluated the combination of treatments as acceptable according to dichotomisation which emphasises the acceptability of combining both HSR and an injection.

Due to loss of training diaries, it is difficult to draw firm conclusions regarding exercise compliance in our study. Exercise compliance is considered a large challenge in the rehabilitation of musculoskeletal conditions, and strategies to increase compliance should be considered whenever exercises are prescribed [35, 36]. Participants performed approximately 75% of the training sessions prescribed which may be interpreted as a high compliance [37]. To increase compliance, we used training diaries and told patients that complying with the exercises was associated with the odds of recovery, but additional strategies such as phone calls or SMS reminders may be needed to increase compliance even further in future trials [35].

### Interpretation of explorative outcomes

This study was not powered to detect changes over time in the explorative outcomes, and the results should be interpreted cautiously; however, several outcome measures pointed in the same direction. Participants improved in FHSQ pain, function, and footwear and in PSEQ from baseline to the 4-week follow-up, but there was only a negligible change from the 4-week to the 8-week follow-up. This is similar to what was observed by both McMillan et al. and Ball et al. following an ultrasound-guided corticosteroid injection [11, 12]. Individuals with plantar fasciopathy who perform HSR will usually experience a steady improvement whereas those who are treated with a corticosteroid injection will experience a fast improvement with no further benefit hereafter [11, 12, 17, 21]. While we did not compare the combination to either of the individual interventions, the trajectory of improvement in our study looks similar to what has been observed in both lateral elbow tendinopathy and gluteal tendinopathy where an injection may hamper the effect of exercises [15, 38]. We only followed participants for 8 weeks as this was sufficient to allow for an evaluation of acceptability, and thus, we cannot make any statement on the long-term outcomes of combing HSR with a corticosteroid injection. Johannsen et al. [18] found a combination of repeated injections and different exercises to be superior to either exercises or injections alone. That study did not include follow-ups between baseline and the 3-month follow-up, so it is not possible to compare our findings with theirs.

To our knowledge, this was the first study that investigated the pain reduction following an ultrasound-guided corticosteroid injection by collecting daily pain data from the days before and after the injection was performed. Contrary to common expectation, the injection did not lead to a large pain reduction within a few days as the reduction of 1.2 NRS is approximately only half of the minimally important difference of NRS in chronic musculoskeletal pain conditions [26, 27]. Clinicians who use injections with corticosteroid for patients with plantar fasciopathy may want to inform patients that they may not experience a pain reduction within the first week.

### Limitations

We believe our study has two limitations. Firstly, we were not able to retrieve five training diaries and two of our feasibility criteria were dependent on data from the diaries. As a result, we cannot draw firm conclusions regarding exercise compliance. In a future trial, additional emphasis should be put on the importance of returning the training diaries when they are handed to participants or other methods for collecting exercise compliance such as SMSs or mobile applications may be used. Secondly, we did not want the exercises to interfere with the measures of daily pain before the injection, so we did not allow participants to start performing the exercise until after the injection. Therefore, some participants did not start the exercise until 10 days after they received the exercise instruction. To counteract this limitation, participants received a written exercise instruction and a link to a video in which the primary investigator showed the instruction.

## Conclusions

Based on participant acceptability and time from participants received the injection to exercise start, combining HSR with an ultrasound-guided corticosteroid injection is feasible and the efficacy compared to other conservative treatments may be investigated in a randomised trial. Due to loss of 5/20 training diaries, firm conclusions regarding exercise compliance could not be drawn.

## Additional file

Additional file 1: Online supplementary table of previous care-seeking behaviour. (DOCX 16 kb)

## Data Availability

Data is available upon request.

## Abbreviations

ANOVA: Analysis of variance
FHSQ: Foot Health Status Questionnaire
GROC: Global Rating of Change
HSR: Heavy-slow resistance training
IPAQ: International Physical Activity Questionnaire
MET: Metabolic equivalent
NRS: Numerical Rating Scale
PSEQ: Pain Self-Efficacy Questionnaire
RM: Repetition maximum
VAS: Visual analogue scale

## References

[1] Landorf KB. Plantar heel pain and plantar fasciitis. BMJ Clin Evid. 2015 Nov 25;2015. PMC4661045 26609884

[2] Buchbinder R (2004). Plantar Fasciitis. N Engl J Med.

[3] Hansen L, Krogh TP, Ellingsen T, Bolvig L, Fredberg U (2018). Long-term prognosis of plantar fasciitis: a 5- to 15-year follow-up study of 174 patients with ultrasound examination. Orthop J Sport Med.

[4] Digiovanni BF, Nawoczenski DA, Malay DP, Graci PA, Williams TT, Wilding GE (2006). Plantar fascia-specific stretching exercise improves outcomes in patients with chronic plantar fasciitis A prospective clinical trial with two-year follow-up. J Bone Joint Surg Am.

[5] Cotchett M, Munteanu SE, Landorf KB, Landorf KB (2016). Depression, anxiety, and stress in people with and without plantar heel pain. Foot ankle Int.

[6] Irving DB, Cook JL, Young MA, Menz HB (2008). Impact of chronic plantar heel pain on health-related quality of life. J Am Podiatr Med Assoc.

[7] Cotchett M, Lennecke A, Medica VG, Whittaker GA, Bonanno DR (2017). The association between pain catastrophising and kinesiophobia with pain and function in people with plantar heel pain. Foot.

[8] Riel H, Vicenzino B, Jensen MB, Olesen JL, Holden S, Rathleff MS. The effect of isometric exercise on pain in individuals with plantar fasciopathy: a randomised crossover trial. Scand J Med Sci Sports. 2018. 10.1111/sms.13296 30203866

[9] Davis PF, Severud E, Baxter DE (1994). Painful heel syndrome: results of nonoperative treatment. Foot Ankle Int.

[10] Babatunde OO, Legha A, Littlewood C, Chesterton LS, Thomas MJ, Menz HB, et al. Comparative effectiveness of treatment options for plantar heel pain: a systematic review with network meta-analysis. Br J Sport Med 2018;bjsports-2017-098998. 10.1136/bjsports-2017-098998 29954828

[11] McMillan AM, Landorf KB, Gilheany MF, Bird AR, Morrow AD, Menz HB (2012). Ultrasound guided corticosteroid injection for plantar fasciitis: randomised controlled trial. BMJ.

[12] Ball EMA, McKeeman HMA, Patterson C, Burns J, Yau WH, Moore OA (2013). Steroid injection for inferior heel pain: a randomised controlled trial. Ann Rheum Dis.

[13] David JA, Sankarapandian V, Christopher PR, Chatterjee A, Macaden AS (2017). Injected corticosteroids for treating plantar heel pain in adults. Cochrane database Syst Rev.

[14] Malliaras P, Barton CJ, Reeves ND, Langberg H (2013). Achilles and patellar tendinopathy loading programmes. Sport Med.

[15] Coombes BK, Bisset L, Brooks P, Khan A, Vicenzino B, H O (2013). Effect of corticosteroid injection, physiotherapy, or both on clinical outcomes in patients with unilateral lateral epicondylalgia. JAMA.

[16] Littlewood C, Bateman M, Brown K, Bury J, Mawson S, May S (2016). A self-managed single exercise programme versus usual physiotherapy treatment for rotator cuff tendinopathy: a randomised controlled trial (the SELF study). Clin Rehabil.

[17] Rathleff MS, Mølgaard CM, Fredberg U, Kaalund S, Andersen KB, Jensen TT (2015). High-load strength training improves outcome in patients with plantar fasciitis: a randomized controlled trial with 12-month follow-up. Scand J Med Sci Sports.

[18] Johannsen FE, Herzog RB, Malmgaard-Clausen NM, Hoegberget-Kalisz M, Magnusson SP, Kjaer M. Corticosteroid injection is the best treatment in plantar fasciitis if combined with controlled training. Knee Surgery, Sport Traumatol Arthrosc. 2018:1–8. 10.1007/s00167-018-5234-6 30443664

[19] Eldridge SM, Chan CL, Campbell MJ, Bond CM, Hopewell S, Thabane L (2016). CONSORT 2010 statement: extension to randomised pilot and feasibility trials. BMJ.

[20] Skovdal Rathleff M, Moelgaard C, Lykkegaard Olesen J (2011). Intra- and interobserver reliability of quantitative ultrasound measurement of the plantar fascia. J Clin Ultrasound.

[21] Riel H, Jensen MB, Olesen JL, Vicenzino B, Rathleff MS. Self-dosed and pre-determined progressive heavy-slow resistance training have similar effects in people with plantar fasciopathy: a randomised trial. J Physiother. 2019. 10.1016/j.jphys.2019.05.011 31204294

[22] Lancaster GA, Dodd S, Williamson PR (2004). Design and analysis of pilot studies: recommendations for good practice. J Eval Clin Pract.

[23] Riel H, Jensen MB, Olesen JL, Rathleff MS (2019). Translation and cultural adaptation of a Danish version of the Foot Health Status Questionnaire for individuals with plantar heel pain. Foot.

[24] Bennett PJ, Patterson C, Wearing S, Baglioni T (1998). Development and validation of a questionnaire designed to measure foot-health status. J Am Podiatr Med Assoc.

[25] Landorf KB, Radford JA (2008). Minimal important difference: Values for the Foot Health Status Questionnaire, Foot Function Index and Visual Analogue Scale. Foot.

[26] Salaffi F, Stancati A, Silvestri CA, Ciapetti A, Grassi W (2004). Minimal clinically important changes in chronic musculoskeletal pain intensity measured on a numerical rating scale. Eur J Pain.

[27] Steroid injections - NHS [Internet]. [cited 2019 Feb 27]. Available from: https://www.nhs.uk/conditions/steroid-injections/

[28] Kamper SJ, Maher CG, Mackay G (2009). Global rating of change scales: a review of strengths and weaknesses and considerations for design. J Man Manip Ther.

[29] Nicholas MK (2007). The pain self-efficacy questionnaire: taking pain into account. Eur J Pain.

[30] Rasmussen MU, Rydahl-Hansen S, Amris K, Samsøe BD, Mortensen EL, Mortensen EL (2016). The adaptation of a Danish version of the Pain Self-Efficacy Questionnaire: reliability and construct validity in a population of patients with fibromyalgia in Denmark. Scand J Caring Sci.

[31] van Poppel MNM, Chinapaw MJM, Mokkink LB, van Mechelen W, Terwee CB (2010). Physical activity questionnaires for adults. Sport Med.

[32] Craig CL, Marshall AL, Sjöström M, Bauman AE, Booth ML, Ainsworth BE (2003). International physical activity questionnaire: 12-country reliability and validity. Med Sci Sports Exerc.

[33] Arain M, Campbell MJ, Cooper CL, Lancaster GA (2010). What is a pilot or feasibility study? A review of current practice and editorial policy. BMC Med Res Methodol.

[34] Billingham SAM, Whitehead AL, Julious SA (2013). An audit of sample sizes for pilot and feasibility trials being undertaken in the United Kingdom registered in the United Kingdom Clinical Research Network database. BMC Med Res Methodol.

[35] Babatunde FO, MacDermid JC, MacIntyre N (2017). A therapist-focused knowledge translation intervention for improving patient adherence in musculoskeletal physiotherapy practice. Arch Physiother.

[36] Jack K, McLean SM, Moffett JK, Gardiner E (2010). Barriers to treatment adherence in physiotherapy outpatient clinics: a systematic review. Man Ther.

[37] Holden MA, Haywood KL, Potia TA, Gee M, McLean S. Recommendations for exercise adherence measures in musculoskeletal settings: a systematic review and consensus meeting (protocol). Syst Rev. 2014;3(1):10. 10.1186/2046-4053-3-10 PMC3923391 24512976

[38] Mellor R, Bennell K, Grimaldi A, Nicolson P, Kasza J, Hodges P (2018). Education plus exercise versus corticosteroid injection use versus a wait and see approach on global outcome and pain from gluteal tendinopathy: prospective, single blinded, randomised clinical trial. BMJ.

