# Supplementary material for: Heavy-slow resistance training in addition to an ultrasound-guided corticosteroid injection for individuals with plantar fasciopathy: a feasibility study
Source: Pilot Feasibility Stud. 2019 Aug 24;5:105. doi: 10.1186/s40814-019-0489-3 (PMC6708237; doi:10.1186/s40814-019-0489-3)
Supplement: Supplementary file 1 — Online supplementary table of previous care-seeking behaviour. (DOCX 16 kb) [file 40814_2019_489_MOESM1_ESM.docx]

| **Online supplementary table of previous care-seeking behaviour** | | |
| --- | --- | --- |
| **Healthcare practitioners seen** (%) | | |
|  | General practitioner  Physiotherapist  None  Medical specialist  Foot therapist  Nurse | 12 (60)  10 (50)  3 (15)  1 (5)  1 (5)  1 (5) |
| **Previous treatments** (%) | | |
|  | Foot orthoses  Strengthening exercises  Activity modification  Pain medication  Stretching  Electrophysical agents  Footwear  Massage  Tape  Acupuncture  Corticosteroid injection  Dietary supplements  Footbath  Elastic bandage | 10 (50)  6 (30)  4 (20)  4 (20)  3 (15)  2 (10)  2 (10)  2 (10)  2 (10)  1 (5)  1 (5)  1 (5)  1 (5)  1 (5) |
| DATA ARE PRESENTED AS COUNT (% of participants (n=20)). | | |
